# Ex-Medicos

**Published:** 1897-03

**Authors:** 


### Ex-Medicos.

About 14 percent of the entire number of medical graduates drop out of the profession within a few years. Some few never practice; others are tempted by better inducements into other fields of work; some are driven to suicide on account of failure; others succumb to contagious diseases; still more lose their health on account of exposure to inclement weather and accident, or on account of mental anxiety. Among those we must include those who become insane or who contract the alcohol, morphine or cocaine habit. Worse than all else, a few are driven into quackery. Any one may make a mistake in the choice of life-work, and it is no discredit to abandon practice. There are plenty of honorable employments for unsuccessful physicians; there are schools to teach, merchandise to sell, drugs to dispense, news to gather; at any rate, there is coal to shovel and wood to saw. It doubtless seems a pity to sacrifice the investment of three or four years' hard work in the study of medicine, but it is cheaper than to sacrifice honor and prostitute medical science to quackery.—A. L. BENEDICT *in Lippincott's.*

